# Influence of a strict glucose protocol on serum potassium and glucose concentrations and their association with mortality in intensive care patients

**DOI:** 10.1186/s13054-015-0959-9

**Published:** 2015-06-22

**Authors:** Esther V. Uijtendaal, Jeannette E.F. Zwart-van Rijkom, Dylan W. de Lange, Arief Lalmohamed, Wouter W. van Solinge, Toine C.G. Egberts

**Affiliations:** Department of Clinical Pharmacy, University Medical Centre Utrecht, PO box 85500, 3508 GA Utrecht, The Netherlands; Division of Pharmacoepidemiology and Clinical Pharmacology, Utrecht Institute for Pharmaceutical Sciences, Faculty of Science, Utrecht University, Utrecht, The Netherlands; Intensive Care Centre, University Medical Centre Utrecht, Utrecht, The Netherlands; Department of Clinical Chemistry and Haematology, University Medical Centre Utrecht, Utrecht, The Netherlands

## Abstract

**Introduction:**

Tight glucose control therapy (TGC) has been implemented to control hyperglycemia in ICU patients. TGC may also influence serum potassium concentrations. We therefore investigated the influence of TGC on both serum glucose and serum potassium concentrations and associated mortality.

**Method:**

We performed a retrospective analysis including all patients admitted to the ICU of a tertiary hospital for 24 hours or more and with at least three serum glucose and serum potassium concentrations between 1999–2001 (conventional period), 2002–2006 (implementation period) or 2007–2009 (TGC period). Segmented regression analysis was used to estimate changes in outcomes that occurred after the intervention controlling for pre-intervention trends. Means and standard deviations (SDs) of serum glucose and serum potassium concentrations, and rate of severe hypoglycemia (≤2.2 mmol/L) and hypokalemia (≤3 mmol/L), were compared between the TGC and conventional period.

**Results:**

Although mean serum glucose concentrations dropped 2.1 mmol/L (95 % CI =−1.8 to −2.3 mmol/L, p<0.002), mean serum potassium concentrations did not change (absolute increase 0.02 mmol/L; 95 % CI = −0.06 to 0.09 mmol/L, p=0.64). The rate of severe hypoglycemia increased with 5.9 % (95 % CI=−3.0 to −8.9, p<0.002), but the rate of hypokalemia remained equal (absolute reduction 4.8 %; 95 % CI = −11.1 % to 1.5 %, p=0.13). The SD of serum glucose concentrations within a patient did not change, while the SD of serum potassium concentrations even decreased 0.04 mmol/L (95 % CI = −0.01 to −0.07, p=0.01). ICU mortality decreased but this decrease was not significant (absolute difference −3.63 %; 95 % CI = −9.33 to 2.09, p=0.20).

Mean serum glucose concentrations, mean serum potassium concentrations and SDs of both serum glucose and serum potassium concentrations were all independently associated with ICU mortality. Highest mortality rates were seen at both the lowest and highest mean values (U/J-shaped association) and mortality rates increased with increasing variability (SDs) for both serum glucose and serum potassium concentrations.

**Conclusion:**

Our study shows that a TGC was not associated with an increased risk of serum potassium related events. Low and high mean values and high variability of both serum glucose and serum potassium concentrations are predictors for high ICU mortality.

**Electronic supplementary material:**

The online version of this article (doi:10.1186/s13054-015-0959-9) contains supplementary material, which is available to authorized users.

## Introduction

Hyperglycemia in response to critical illness has been associated with increased morbidity and mortality [1]. The mechanism suggested for this increased risk is that elevated glucose concentrations increase the concentration of several toxic intracellular derivatives that are generated as by-products of the glycolytic pathway [2, 3]. Especially during severe illness, the expression of insulin-independent glucose transporters on the membranes of several cell types is upregulated, which may allow high circulating glucose concentrations to overload and damage these cells [4–7]. Based upon this line of reasoning, Van den Berghe et al. investigated whether a tight glycaemic control (TGC) protocol (target serum glucose concentration 4.4−6.1 mmol/L (80–110 mg/dL)) would reduce mortality in ICU patients [8]. In this landmark clinical trial absolute mortality was reduced by 3.4 %. This led to the implementation of TGC protocols in many ICUs worldwide.

Subsequent studies performed in medical ICU patients, however, failed to reproduce the reduction in mortality [9–11]. The NICE-sugar investigators even reported increased mortality when TGC was compared to conventional treatment [12]. Since then, TGC has become a major area of debate among medical specialties involved in the care of acutely ill patients. Several hypotheses have been postulated to explain the contradictory results.

First, the characteristics of study populations differed between the different clinical trials that were carried out. TGC seems to benefit surgical ICU patients more than medical ICU patients [13]. In the setting where hyperglycemia is triggered by surgery, the delay between onset of hyperglycemia and the start of glycemic control is short. Medical ICU patients may have suffered from chronic diseases and hyperglycemia before ICU admission and time from the onset of symptoms to the start of TGC may be longer. As such the TGC protocol may be more beneficial in surgical ICU patients. The most recent meta-analysis, however, showed that there is no significant benefit of TGC in either medical or surgical patients [14]. Second, a TGC protocol can be expected to increase the incidence of severe hypoglycemia, which in itself raises the risk of mortality. Increased incidence of severe hypoglycemia (≤2.2 mmol/L (40 mg/dL) serum glucose concentration) was seen in all studies that applied a TGC protocol [15]. The highest rates of severe hypoglycemia were generally found in studies that applied the lowest serum glucose targets, varying, however, with the complexity of the TGC protocols [16]. An increased rate of severe hypoglycemia may also reflect a large fluctuation of serum glucose concentrations implying that these vulnerable patients may also be exposed to high glucose concentrations, although mean serum glucose concentrations are relatively low [17]. In vitro studies showed that a large variability in glucose concentrations may enhance cell apoptosis [18, 19] and some clinical studies confirmed an independent association between large variations in serum glucose concentrations and mortality [20–23]. Third, in addition to serum glucose, serum potassium may also be a significant factor influencing patients’ outcomes. It is well known that insulin induces a shift of potassium from the extracellular to the intracellular compartment. As a result, the implementation of a TGC protocol may also induce larger variations in serum potassium concentrations and more frequently hypokalemia and associated complications such as arrhythmia. In the NICE-sugar study there was an increased risk of cardiovascular deaths in the TGC-group, but its cause remained unclear [12]. It was suggested that a missed episode of arrhythmogenic hypokalemia may have contributed to the excess of cardiovascular deaths [24], although, arrhythmias caused only 83 out of 1,580 deaths and there was no difference in the risk of death from arrhythmia in the two groups (relative risk (RR) for intensive versus conventional control of 1.1; 95 % CI 0.7, 1.7, *p* = 0.6 [25, 26]. There are studies that have reported increased incidence of hypokalemia, and emphasized the necessity for combined glucose and potassium monitoring to prevent hypokalemia-induced arrhythmia [24, 27]. Other studies did not mention the measurement of serum potassium concentrations as a part of the TGC protocol.

Because of the ongoing discussion, we investigated in our own academic ICU what the influence was of the implementation of a TGC protocol on the rate of severe hypoglycemia and hypokalemia and the variation in serum glucose and potassium concentrations. Furthermore, the association between mortality and both serum glucose and potassium concentrations and variability was studied during the TGC period.

## Methods

### Setting and study population

This retrospective observational study was carried out at the 32-bed ICU of the University Medical Centre Utrecht (UMCU), which is a tertiary care teaching hospital in the Netherlands. Surgical, internal, neurological and cardiothoracic ICU patients are treated on this ICU. A TGC policy was consecutively implemented on all specialties of the ICU during the years 2002–2006. These years were defined at the implementation period. The years before implementation, 1999–2001, were defined as the conventional period and the years 2007–2009 were defined as the TGC period.

During the conventional period, conventional serum glucose control was applied to all patients, which implied that insulin therapy was started if the serum glucose concentrations were above 12 mmol/L (215 mg/dL) and that serum glucose concentrations were maintained at 10–11 mmol/L (180–200 mg/dL). During the TGC period, a TGC protocol was followed, aiming to maintain serum glucose concentrations at 4.4–6.1 mmol/L (80–110 mg/dL). Details of this TGC protocol can be found in Additional file 1. In the conventional period, the policy was to start potassium supplementation if serum potassium was <4 mmol/L; this policy did not change in the TGC period. After 2009, target glucose concentrations were gradually increased to <8 mmol/L; this period is not included in the analysis. Guidance on glucose and potassium concentrations was paper based, not computer assisted.

**Fig. 1:**
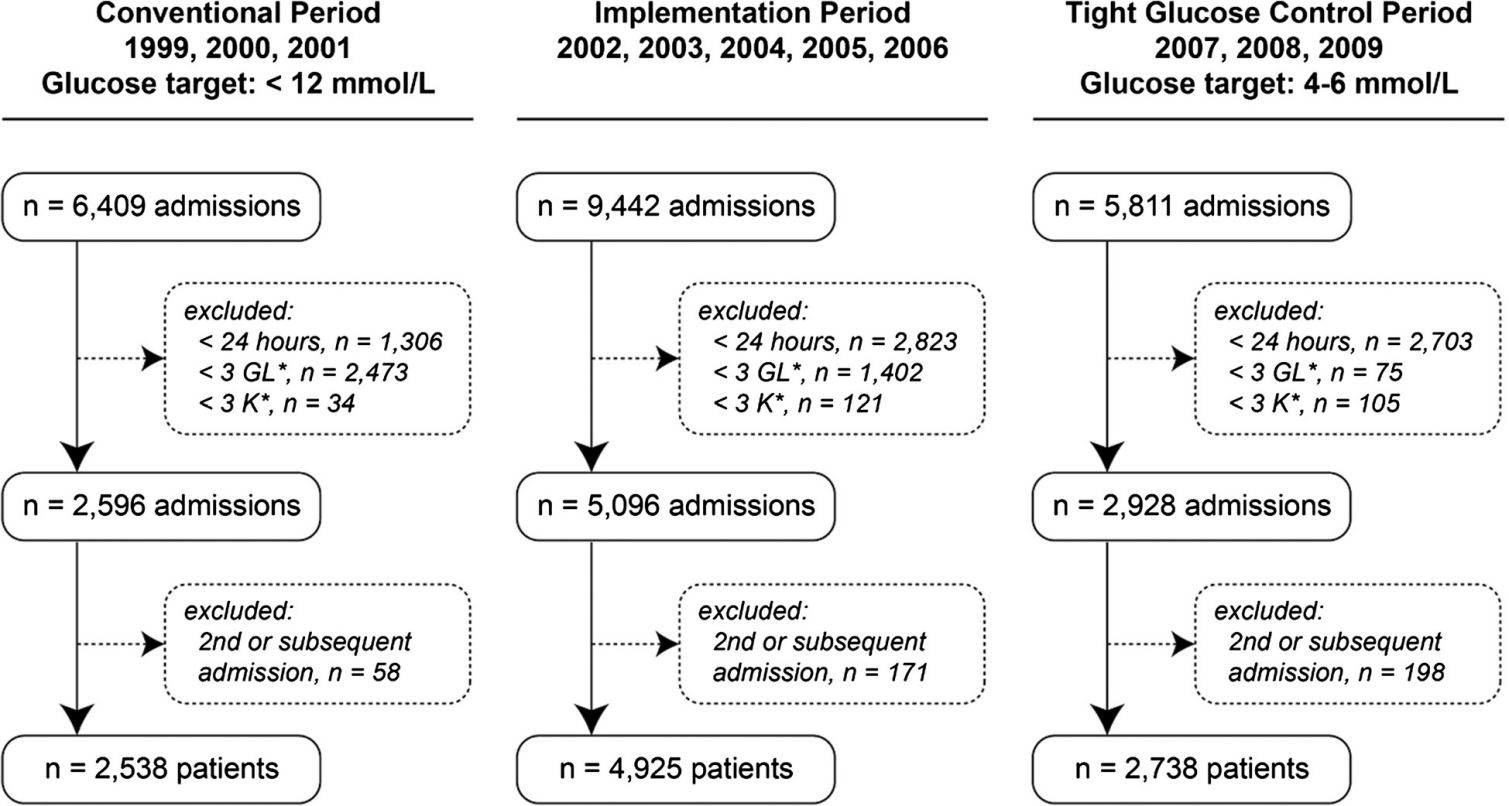
Inclusion of study patients

Adult patients (≥18 years) who were admitted to the ICU for more than 24 hours in the years 1999–2009 were eligible for inclusion. To be able to calculate serum glucose and potassium variability, patients with fewer than three valid serum glucose or fewer than three valid serum potassium measurements during ICU admission were excluded. Moreover, if a patient was admitted to the ICU more than once, only the first admission was included (Fig. 1).

Multiple machines, including point of care meters for blood glucose, were used to measure glucose and potassium concentrations throughout the study. Point of care meters for blood glucose did not provide potassium readings. The machines were all calibrated to each other. For research purposes all laboratory values are routinely exported to the Utrecht Patient Oriented Database (UPOD). UPOD is an infrastructure of relational databases comprising data on patient demographics, hospital discharge diagnoses, medical procedures, medication orders and laboratory tests for all patients treated at the UMCU, and has been described in detail elsewhere [28]. The anonymized data analysis in this study was performed in accordance with the guidelines and Dutch legislation and it was approved by the medical ethical committee of our institution (Medical Ethical Review Committee University Medical Centre Utrecht, MERC WAG/om/14/013125). As only routinely documented patient data were used in this study, the ethical board of the University Medical Centre Utrecht waived the need for informed consent.

### Outcomes

Four outcome parameters were determined for each individual patient:

1. mean serum glucose and potassium concentrations: means of serum glucose and serum potassium concentrations were calculated for each patient during ICU stay
2. severe hypoglycemia and hypokalemia: proportion of patients with at least one severe hypoglycemic episode and proportion of patients with at least one hypokalemic episode. Severe hypoglycemia was defined as a serum glucose measurement ≤2.2 mmol/L and hypokalemia was defined as a serum potassium measurement ≤3.0 mmol/L
3. variability of serum glucose and serum potassium concentrations: mean SD of individually calculated SDs of serum glucose and serum potassium.
4. ICU mortality.

### Data analysis

First, interrupted time series analysis was performed to compare the TGC period with the conventional period. Patient characteristics were compared using the *t* test (mean values), the chi-square test (proportions) and Mann–Whitney *U* test (median values). Segmented regression analysis was used to estimate changes in outcomes that occurred after the intervention controlling for pre-intervention trends [29]. The intervention was the implementation of a TGC protocol between January 2002 and January 2007. Outcome values during the implementation period were modelled as a separate segment. Each time point in the time series data represents a 3-month period resulting in the recommended minimum of 12 time points before and 12 time points after the intervention [29]. The following linear regression model was used:

1 $$ {\mathrm{Y}}_{\mathrm{t}}={\beta}_0+{\beta}_1*\ {\mathrm{t}\mathrm{ime}}_1+{\beta}_2*\ {\mathrm{t}\mathrm{ime}}_2+{\beta}_3*\ {\mathrm{t}\mathrm{ime}}_3 $$

where Y_t_ = mean serum glucose concentration at time = t; β_0_ = intercept = mean serum glucose concentration at time = 0; β_1_ = baseline trend = change in serum glucose concentration during the conventional period; time_1_ = time after start of the conventional period (Jan 1, 1999); β_2_ = trend change during the implementation period, adjusted for the baseline trend; time_2_ = time after start of the implementation period (Jan 1, 2002); β_3_ = trend change during the TGC period, adjusted for the baseline trend and trend change during the implementation period; time_3_ = time after start of the TGC Period (Jan 1, 2007).

The change in mean serum glucose values between the end of the control period and the start of the TGC period are calculated from:

2 $$ \mathrm{Change} = \left({\beta}_{1 + }{\beta}_2\right)\ *\ \mathrm{duration}\ \mathrm{of}\ \mathrm{implementation} $$

The same regression model was used for the proportion of patients with severe hypoglycemia, the mean SDs for serum glucose concentrations, mean serum potassium concentrations, proportion of patients with hypokalemia, mean SDs for serum potassium concentrations and ICU mortality.

Second, the relationship between mean serum glucose concentrations, mean serum potassium concentrations and ICU mortality was studied. This relationship was also studied for the SD of serum glucose, SD of serum potassium concentrations and mortality. For this analysis, only patients who either survived or who died on the ICU during the TGC period were included. Logistic regression analysis was performed to determine the association between mean glucose concentrations and risk of ICU mortality. Adjustments were made for mean serum potassium concentrations, age and gender. As adjustment for length of stay may be influenced by the TGC therapy, it is left out of the adjustment [26]. A similar procedure was performed to determine the association between mean serum potassium concentrations (with adjustments for mean serum glucose concentrations, age and gender), SD of serum glucose concentrations and SD of serum potassium concentrations and ICU mortality. Moreover, to study the relationship between the combined mean serum glucose and mean serum potassium concentrations on ICU mortality, patients were divided into eight equal subgroups for their mean serum glucose and eight equal subgroups for their mean serum potassium values. This resulted in 64 possible mean glucose-mean potassium subgroup-combinations. ICU mortality was calculated for each combination.

Time series analysis was performed using SAS 9.2, SAS Institute Inc, Cary, NC, USA using the PROC REG procedure. All other data analyses were performed using SPSS release 20 (SPSS release 20 (SPSS, Inc., Chicago, IL, USA).

## Results

During the study period 21,662 admissions were available for selection. After application of the inclusion criteria, 2,538 patients were eligible for inclusion in the conventional period, 4,925 in the implementation period and 2,730 in the TGC period (Fig. 1). Baseline characteristics of the patients are shown per period in Table 1. Compared to patients included in the conventional period, patients in the TGC period were older (59.6 vs 57.6 years), mean serum creatinine concentrations were lower (112 vs 121 mmol/L), the length of ICU and hospital stay was shorter (3.1 vs 4.5 and 16 vs 18 days respectively) and the number of patients admitted due to an emergency was lower (45 vs 54 %). ICU mortality was also lower in the TGC period (14.4 vs 18.6 %). The median number of serum glucose measurements per patient per ICU day increased approximately 4.7 times (*p* <0.002) after implementation of the TGC protocol. The median number of serum potassium measurements per patient per ICU day, increased from 2.3 measurements to 2.8 (*p* <0.002).

**Table 1:** Baseline characteristics

	Control period 1999, 2000, 2001 (n = 2,538)	Implementation period 2002, 2003, 2004, 2005, 2006 (n = 4,925)	Tight glucose control period 2007, 2008, 2009 (n = 2,730)	*P* value
Male gender, %	60.3	63.1	60.7	0.782^a^
Age, years, mean (95 % CI)	57.6 (57.0, 58.3)	59.9 (59.4, 60.3)	59.6 (59.0, 60.2)	<0.002^b^
Length of stay in ICU, days, median (IQR)	4.5 (2.0, 11.3)	2.8 (1.1, 8.2)	3.1 (1.8, 8.3)	<0.002^c^
Length of stay hospital, days, median (IQR)	18.0 (9.0, 38.7)	14.0 (7.9, 30.5)	16.0 (8.1, 32.8)	<0.002^c^
Hospitalization due to emergency, %	54.0	41.8	45.1	<0.002^a^
Died on ICU, number (%)	472 (18.6)	659 (13.4)	393 (14.4)	<0.002^a^
Died in hospital, number (%)	135 (5.3)	275 (5.6)	147 (5.4)	0.741^a^
Number of glucose measurements per patient, median (IQR)	8 (5–18)	12 (6–37)	27 (13–76)	<0.002^c^
Number of glucose measurements per patient per ICU day, median (IQR)	1.9 (1.2, 3.3)	4.8 (2.8, 7.8)	8.9 (7.3, 10.6)	<0.002^c^
Number of potassium measurements per patient, median (IQR)	10 (6–21)	9 (6–17)	9 (6–20)	<0.002^c^
Number of potassium measurements per patient per ICU day, median (IQR)	2.3 (1.5, 3.5)	2.8 (1.9, 4.8)	2.8 (2.0, 3.9)	<0.002^c^
pH, mean (95 % CI)	7.42 (7.38, 7.45)^†^	7.39 (7.39, 7.39)^†††††^	7.39 (7.39, 7.39)^††^	0.125^b^
Creatinine, mean (95 % CI)	120.6 (115.7, 125.4)^†††^	108.5 (105.6, 111.4) ^††††††^	111.5 (107.5, 115.5)^††††^	<0.002^b^

*P* values represent differences between control period and tight glucose control period. ^a^Chi square test; ^b^independent samples *t* test; ^c^Mann-Whitney *U* test; ^†^0.6 % missing; ^††^8.6 % missing; ^†††^0.1 % missing; ^††††^0.7 % missing; ^†††††^0.6 % missing; ^††††††^0.5 % missing

### Comparison of the TGC period with the conventional period

**Fig. 2:**
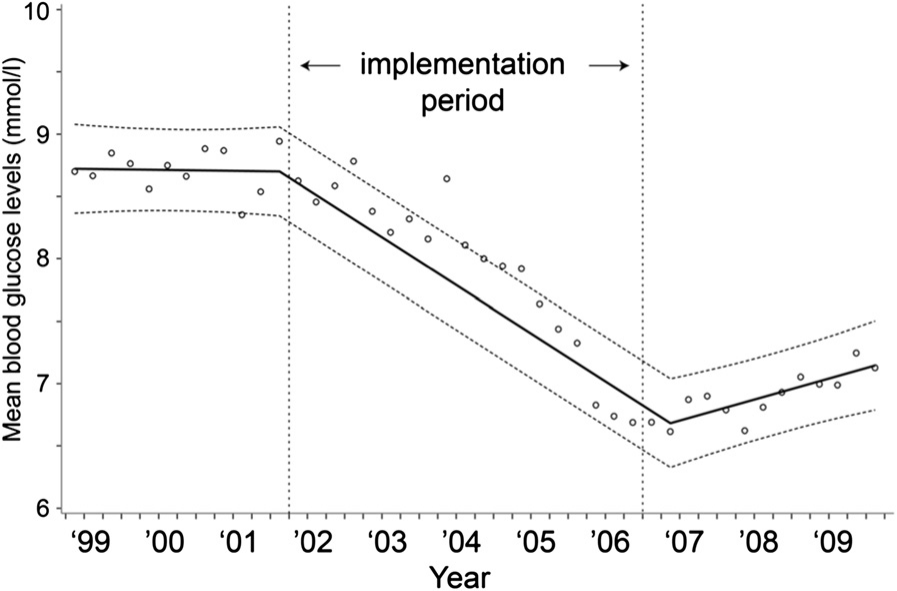
Mean serum glucose concentrations during the study period. y = 8.720875 - 0.00002*time1 - 0.0011*time2 + 0.001605*time3

**Fig. 3:**
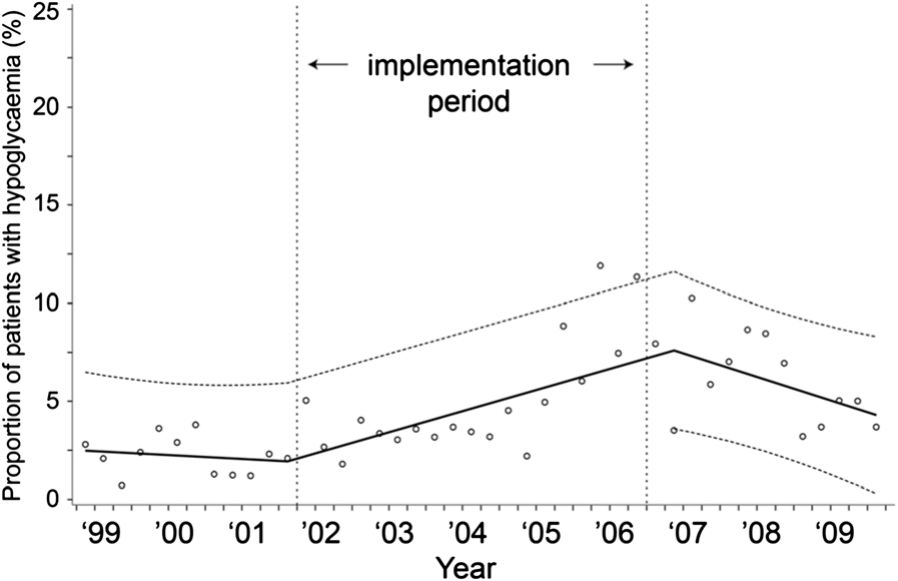
Percentage of patients with a period of severe hypoglycemia (≤2.2 mmol/L) during the study period. y = 2.268667 - 0.00056*time1 + 0.003807*time2 - 0.00659*time3

**Fig. 4:**
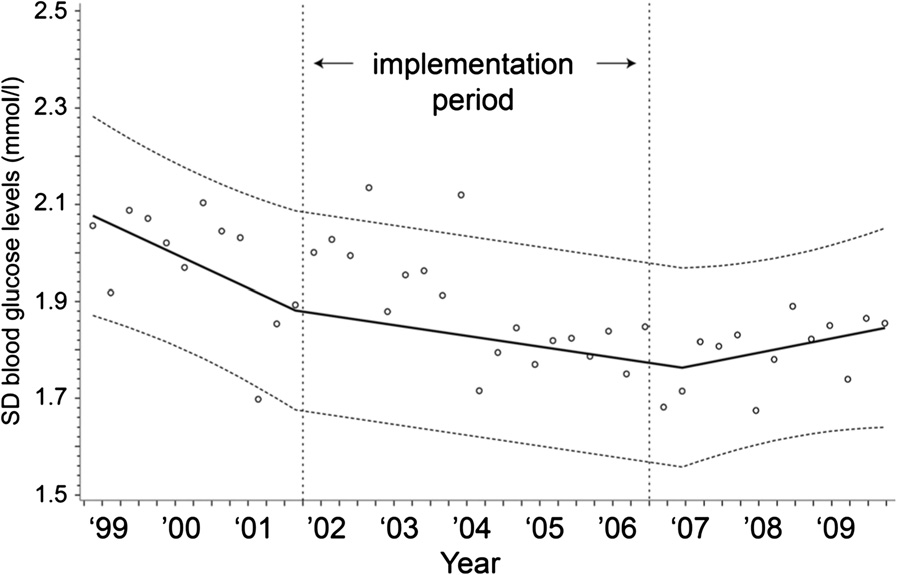
Means of individual standard deviations for serum glucose concentrations during the study period. y = 2.085461 - 0.00019*time1 + 0.000133*time2 + 1.342228*time3

The mean serum glucose concentrations, proportion of patients with a severe hypoglycemia and SD values for serum glucose concentrations over time are displayed in Figs. 2, 3 and 4. After implementation of the TGC protocol, mean serum glucose concentrations dropped from 8.7 to 6.6 mmol/L, an absolute reduction of 2.1 mmol/L (95 % CI = −1.8, −2.3, *p* <0.002, Fig. 2). The percentage of patients with a severe hypoglycemia increased from 1.7−7.6 %, an absolute increase of 5.9 % (95 % CI = 3.0, 8.9 %, *p* <0.002). The variability of serum glucose concentrations within patients did not change after implementation of the TGC protocol (absolute difference −0.11 mmol/L; 95 % CI = −0.26, 0.03, *p* = 0.13).

**Fig. 5:**
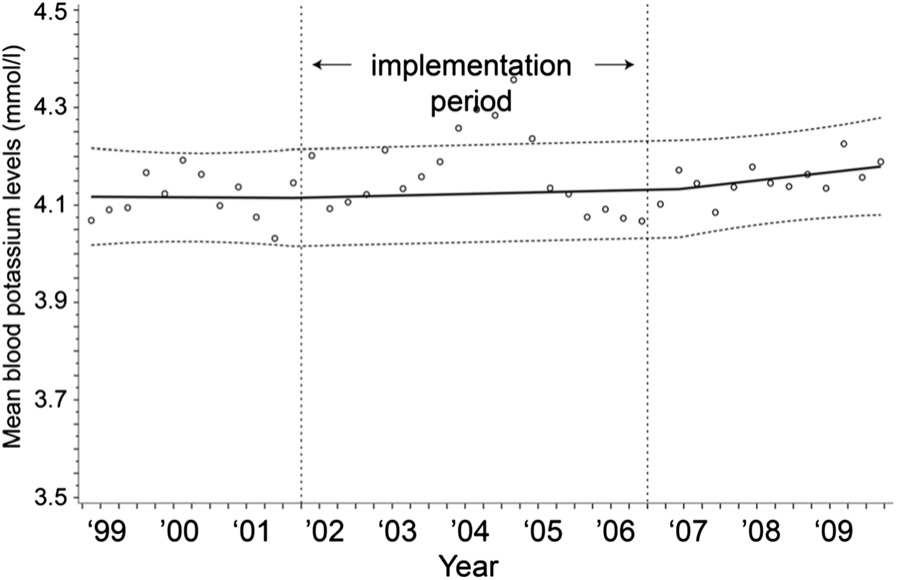
Mean serum potassium concentrations during the study period. y = 4.117654 - 0.0000025*time1 + 0.0000114*time2 + 0.0000374*time3

**Fig. 6:**
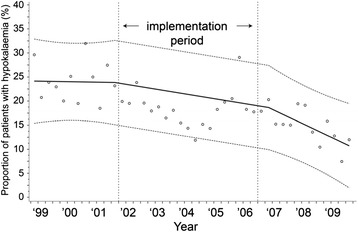
Percentage of patients with a period of hypokalemia (≤3 mmol/L) during the study period. y = 24.14247 - 0.00029*time1 - 0.00236*time2 - 0.004526*time3

**Fig. 7:**
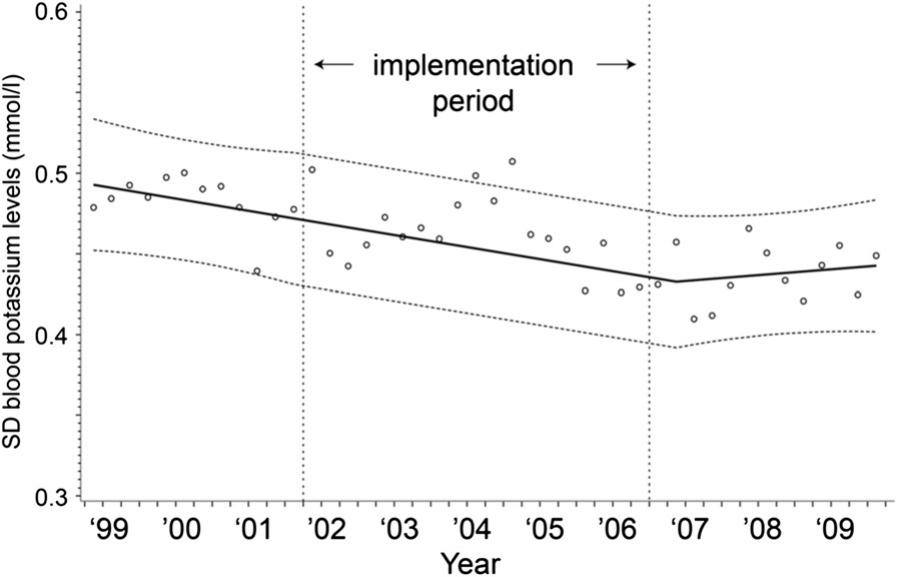
Means of individual standard deviations for serum potassium concentrations during the study period. y = 0.493879 - 0.000021*time1 - 0.000033*time2 - 0.0000311*time3

Similarly to serum glucose, values over time for serum potassium are displayed in Figs. 5, 6, and 7. Mean serum potassium concentrations stayed the same (absolute increase 0.02 mmol/L, 95 % CI = −0.06, 0.09 mmol/L, *p* = 0.64). The percentage of patients with hypokalemia dropped from 23.8 to 19.0 %, an absolute reduction of 4.8 % (95 % CI = −11.1, 1.5 %, *p* = 0.13), however, this reduction was not statistically significant. The variability of serum potassium concentrations within a patient decreased after implementation of the TGC; SD values decreased by 0.04 mmol/L (95 % CI = −0.01, −0.07, *p* = 0.01).

**Fig. 8:**
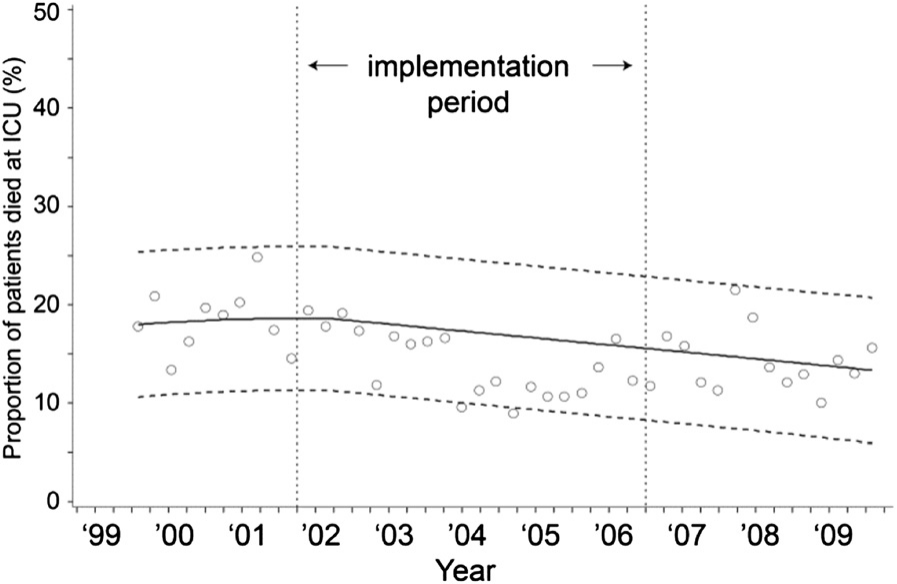
ICU mortality during the study period. y = 18.3116 + 0.000695*time1 - 0.00268*time2 + 0.0000777*time3

ICU mortality over time is displayed in Fig. 8. ICU mortality decreased from 19.0−15.4 %, but this decrease was not significant (95 % CI=−9.3, 2.1, *p* = 0.20).

### Relationship between serum glucose and potassium concentrations and ICU mortality during TGC

**Fig. 9:**
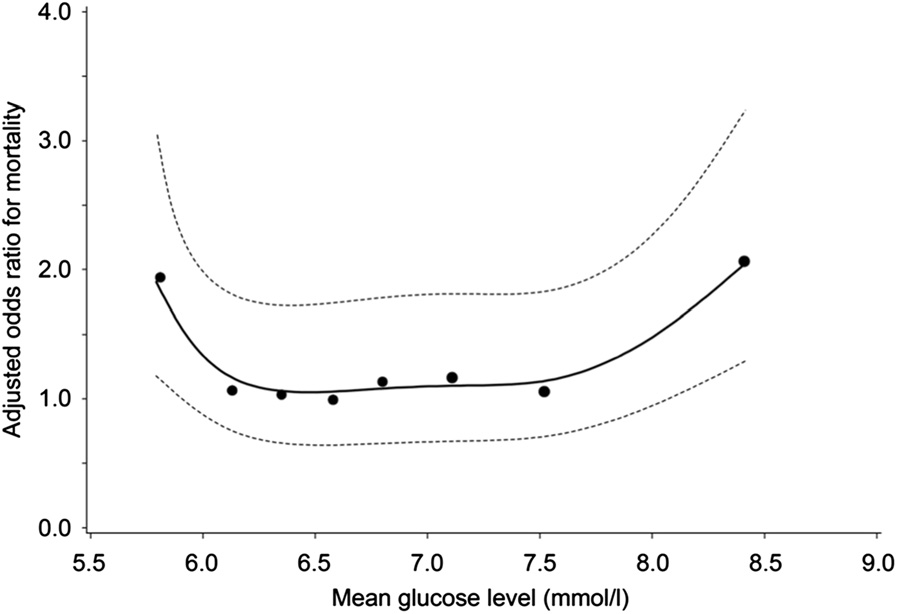
Relationship between mean serum glucose concentrations and adjusted* ODDs ratio on ICU mortality during TGC period. * adjusted for age, gender, length of ICU stay and mean serum potassium concentrations

**Fig. 10:**
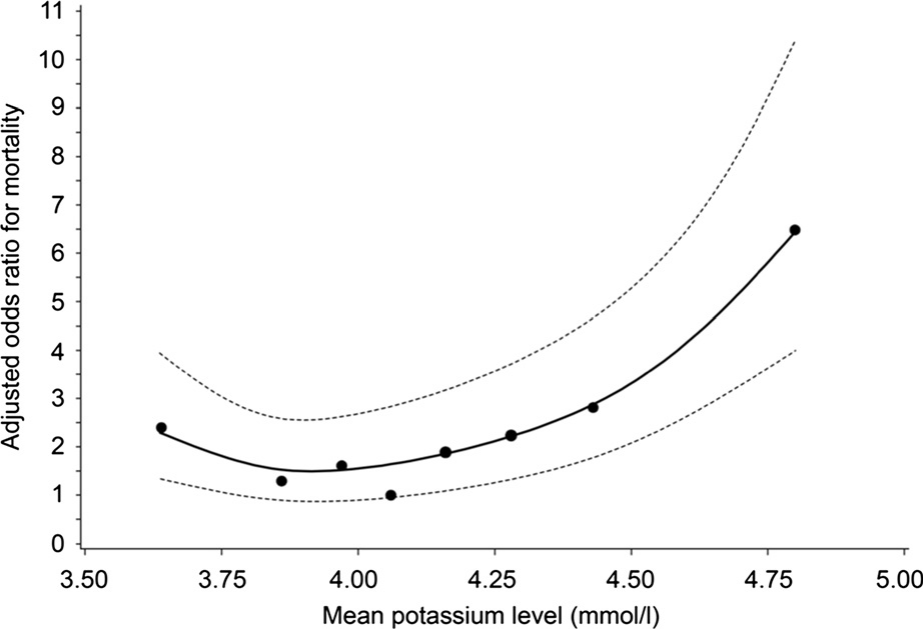
Relationship between mean serum potassium concentrations and adjusted* ODDs ratio on ICU mortality during TGC period. * adjusted for age, gender, length of ICU stay and mean serum glucose concentrations

During TGC, overall ICU mortality was 15.2 %. Multivariate logistic regression analysis revealed a U-shaped relationship between mean serum glucose concentrations and mortality (Fig. 9). This association was also found in the conventional and implementation period. A J-shaped relationship was found between serum potassium concentration and mortality (Fig. 10). Both trends were statistically significant and independent of age and gender, and mean serum potassium or mean serum glucose concentrations, respectively.

**Fig. 11:**
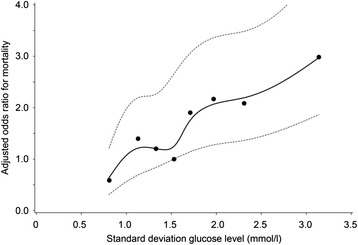
Relationship between SD for serum glucose concentrations and adjusted* ODDs ratio on ICU mortality during TGC period. * adjusted for age, gender, length of ICU stay and SD for serum potassium concentrations

**Fig. 12:**
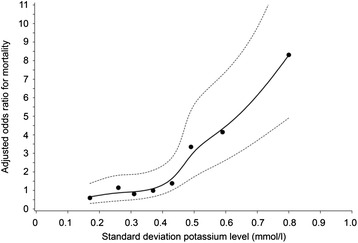
Relationship between SD for serum potassium concentrations and adjusted* ODDs ratio on ICU mortality during TGC period. * adjusted for age, gender, length of ICU stay and SD for serum glucose concentrations

Mortality increased with increasing variability (SDs) of both serum glucose and serum potassium concentrations (Figs. 11 and 12). These trends were also independent of age and gender and SD for serum potassium or SD for serum glucose concentrations, respectively.

The percentages of patients who died at the ICU in each combined mean serum glucose and mean serum potassium concentration subgroup are displayed in Table 2. When stratified for both mean serum glucose and mean serum potassium concentrations, extremes in mortality were seen in both the combined highest mean serum potassium and lowest serum glucose subgroup (45.2 %) and the combined highest mean serum glucose and lowest mean serum potassium subgroup (40.5 %). ICU mortality increased with increasing variability of both serum glucose and serum potassium concentrations with extremes at the highest SDs of the combination (Table 3).

**Table 2:** ICU mortality (percentage and numbers) per stratum of mean serum glucose and mean serum potassium concentrations during the TGC period

	Average potassium		1 (2.78-3.77)	2 (3.78-3.91)	3 (3.92-4.00)	4 (4.01-4.10)	5 (4.11-4.21)	6 (4.22-4.3)	7 (4.35-4.57)	8 (4.58-6.04)	
			n=317	n=339	n=307	n=334	n=320	n=311	n=336	n=319	Total
Average glucose	1 (3.18-6.00)	n=319									65/319 (20.4 %)
	2 (6.01-6.25)	n=322									44/322 (13.7 %)
	3 (6.26-6.46)	n=338									45/338 (13.3 %)
	4 (6.47-6.67)	n=304									36/304 (11.8 %)
	5 (6.68-6.93)	n=331									45/331 (13.6 %)
	6 (6.94-7.29)	n=332									45/322 (14.0 %)
	7 (7.30-7.85)	n=325									41/325 (12.6 %)
	8 (7.86-29.98)	n=322									72/322 (22.4 %)
Total		n=2583	44/317 (13.9 %)	29/339 (8.6 %)	34/307 (11.1 %)	26/334 (7.8 %)	44/320 (13.8 %)	48/311 (15.4 %)	61/336 (18.2 %)	107/319 (33.5 %)	393/2583 (15.2 %)

**Table 3:** ICU mortality (percentage and numbers) per stratum of standard deviation of serum glucose and serum potassium concentrations during the TGC period

	SD potassium		1 (0.00-0.22)	2 (0.23-0.28)	3 (0.29-0.34)	4 (0.35-0.39)	5 (0.40-0.45)	6 (0.46-0.53)	7 (0.54-0.66)	8 (0.67-2.11)	
			n=323	n=291	n=370	n=298	n=330	n=312	n=337	n=322	Total
SD glucose	1 (0.00-0.99	n=318									12/318 (3.8 %)
	2 (1.00-1.23)	n=330									34/330 (10.3 %)
	3 (1.24-1.42)	n=318									33/318 (10.4 %)
	4 (1.43-1.61)	n=336									32/336 (9.5 %)
	5 (1.62-1.81)	n=311									57/311 (18.3 %)
	6 (1.82-2.12)	n=322									68/322 (21.1 %)
	7 (2.13-2.60)	n=323									66/323 (20.4 %)
	8 (2.61-24.45)	n=325									91/325 (28.0 %)
Total		n=2583	12/323 (3.7 %)	20/291 (6.9 %)	20/370 (5.4 %)	19/298 (6.4 %)	33/330 (10.0 %)	67/312 (21.5 %)	89/337 (26.4 %)	133/322 (41.3 %)	393/2583 (15.2 %)

## Discussion

Although implementation of TGC resulted in a significant absolute reduction of mean serum glucose concentrations of 2.1 mmol/L, mean serum potassium concentrations did not change significantly. The proportion of patients with a severe hypoglycemia increased from 1.7 to 7.6 % while the proportion of patients with a hypokalemia did not change. The variability of serum glucose concentrations within patients did not change while the variability of serum potassium values decreased. TGC did not change ICU mortality significantly. The highest mortality was seen in patients with low serum glucose concentrations combined with high serum potassium concentrations and in patients with high serum glucose concentrations combined with low serum potassium concentrations. ICU mortality increased with increasing variability for both serum glucose and serum potassium concentrations.

Implementation of a TGC led to 4.7 times more frequent measurement of serum glucose concentrations per ICU day (8.9 vs 1.9), lower mean glucose concentrations and higher incidence of severe hypoglycemia. As both moderate and severe hypoglycemia are associated with increased risk of death, elevated rates of both moderate and severe hypoglycemia should be prevented [26, 30]. In our setting, after implementation of the TGC protocol, the incidence of severe hypoglycemia was 7.6 %. This percentage compares favourably to the percentages 5.1 – 28.6 % found in other studies [8, 11, 15]. As it is known that spontaneous hypoglycemia is correlated with higher mortality than hypoglycemia occurring during insulin administration [31], it would have been interesting to know whether the increase of severe hypoglycemia could be assigned to insulin administration. Unfortunately, data on insulin administration were not available. Moreover, it is hard to unravel whether the increased incidence of severe hypoglycemia is caused by the lower mean glucose concentrations or whether it is caused by the more frequent measuring of serum glucose concentrations, as more frequent monitoring may also increase the chance to detect severe hypoglycemia.

As insulin therapy induces a shift of potassium from the extracellular to the intracellular space, we expected that the proportion of patients with hypokalemia would increase. However, in our study mean serum potassium concentrations and the proportion of patients with hypokalemia did not change significantly after implementation of the TGC protocol. One study did find increased incidence of hypokalemia after implementation of TGC, but this study used a higher threshold to define hypokalemia [24, 32]. Another study reported an increase of patient days with hypokalemia just after application of TGC, but this increase did not persist throughout the study period [27]. We believe that a serum potassium concentration ≤3 mmol/L represents a more appropriate threshold as this comprises a serious risk of arrhythmia [33]. Apparently, the unchanged policy in our setting to maintain serum potassium concentrations >4 mmol/L was successful in preventing severe hypokalemia. The exact extent of potassium supplementation, however, is not known, which complicates the interpretation of this finding. One may also speculate that after TGC, potassium can shift back from the intracellular to the extracellular cellular space and thereby increase mortality. Although we cannot exclude this possibility, our results do not support this hypothesis.

Some studies showed that TGC increased the variability of serum glucose concentrations. This is of clinical importance as an increased variability is associated with a rise in mortality. In our study, after implementation of TGC, serum glucose SD values stayed about equal. This means that TGC can be implemented in the general practice without an increase of serum glucose SD values. The SD of serum potassium concentrations even decreased, while the target concentrations and monitoring frequency remained unchanged.

As was shown in the follow up of the Leuven study in critically ill children, a possible effect of TGC is a reduction in ICU mortality, despite an increase in severe hypoglycaemia [34]. In line with these findings, ICU mortality in our study decreased from 19.0 to 15.4 %, but this reduction was not significant when it was controlled for the pre-intervention trend in ICU mortality (absolute difference −3.6; 95 % CI = −9.3, 2.1, *p* = 0.20). This means that factors other than TGC have also contributed to the reduction in ICU mortality. As such, we cannot conclude that the implementation of TGC resulted in lower mortality, even though this study was not designed to estimate the effect of TGC on ICU mortality.

During TGC, the highest ICU mortality was seen at both low and high mean serum glucose concentrations. A similar relationship between mean glucose concentrations and mortality was recently described for cardiac patients admitted to the ICU [35]. Mortality, however, was also high at both low and high serum potassium concentrations. A similar relationship between serum potassium concentrations and hospital mortality has recently been described by Hessels et al. [36]. In our study, however, ICU mortality was adjusted for both mean serum glucose and mean serum potassium concentrations. This means that both mean serum glucose and serum potassium concentrations were independently associated with ICU mortality. Moreover, mortality for low and high mean serum potassium concentrations was higher than for low and high mean serum glucose concentrations. Apparently, the association between mean serum potassium concentrations and ICU mortality was stronger than the association between serum glucose concentrations and ICU mortality.

The combined mean serum glucose–mean serum potassium concentrations Table 2 reveals the highest mortality rates in both the combined lowest serum glucose and highest serum potassium subgroup (45.2 %) and the combined highest serum glucose and lowest serum potassium subgroup (40.5 %). Patients in the first subgroup, may have been severely ill, which is often accompanied with high serum potassium concentrations. When these high serum potassium concentrations were treated with insulin, severe hypoglycemia may have resulted. In the latter subgroup, the TGC protocol might have contributed to the high mortality, as correction of the high serum glucose concentration in combination with the relatively low serum potassium concentration may have resulted in unintended hypokalemia.

ICU mortality also increased with increasing SD values for both serum glucose and serum potassium concentrations and the highest mortality rates were seen in the combined highest serum glucose SD and serum potassium SD subgroups. Xu et al. recently also showed a relationship between variability of serum sodium concentrations and mortality, suggesting that high variability is unfavorable for a patient’s outcome [37]. High variability may have been caused by the TGC protocol but may also be the result of the severity of disease. As no information was available about the severity of illness, it was not possible to unravel the association between variability, severity of illness and mortality, which limits the causal interpretation.

Another limitation is that no information was available about the individual use of insulin, parenteral feeding or co-medication. The use of co-medication might have been relevant as it is known that many medications may influence both serum glucose and/or serum potassium concentrations [38–41]. Beta2-sympathicomimetics, for example, may induce hypokalemia and beta_2_-sympathicomimetics are widely used in the ICU [42]. The use of co-medications, however, is not supposed to vary between the conventional and TGC period to an extent that would have influenced the outcome measures.

## Conclusion

In conclusion, our study shows that the TGC protocol that was implemented was not associated with increased risk of serum-potassium-related events. A reduction in ICU mortality due to TGC could not be confirmed. During TGC, both low and high serum glucose and low and high mean serum potassium concentrations are predictors of a high ICU mortality rate. The same applies for high variability in both serum glucose and serum potassium concentrations.

## Key messages

- Implementation led to 4.7 times more frequent measurement of glucose concentrations but the number of potassium measurements increased only 1.2 times.
- Both low and high mean glucose concentrations and low and high mean potassium concentrations predicted a high mortality rate in ICU patients. The same applies for the variability of glucose and potassium concentrations.
- The TGC protocol that was implemented was not associated with increased risk of hypokalemia.

## Additional file

Additional file 1:
**Details of the tight glucose protocol.**

## Abbreviations

MAG: Mean absolute glucose per hour.
SAS: Statistical Analysis System
SPSS: Statistical Package for the Social Sciences
TGC: tight glucose control
UMCU: University Medical Center Utrecht
UPOD: Utrecht Patient Oriented Database
