# Supplementary figures and images for: Influence of a strict glucose protocol on serum potassium and glucose concentrations and their association with mortality in intensive care patients
Source: Crit Care. 2015 Jun 22;19(1):270. doi: 10.1186/s13054-015-0959-9 (PMC4548912; doi:10.1186/s13054-015-0959-9)

Additional file 1: details of the Tight Glucose Protocol

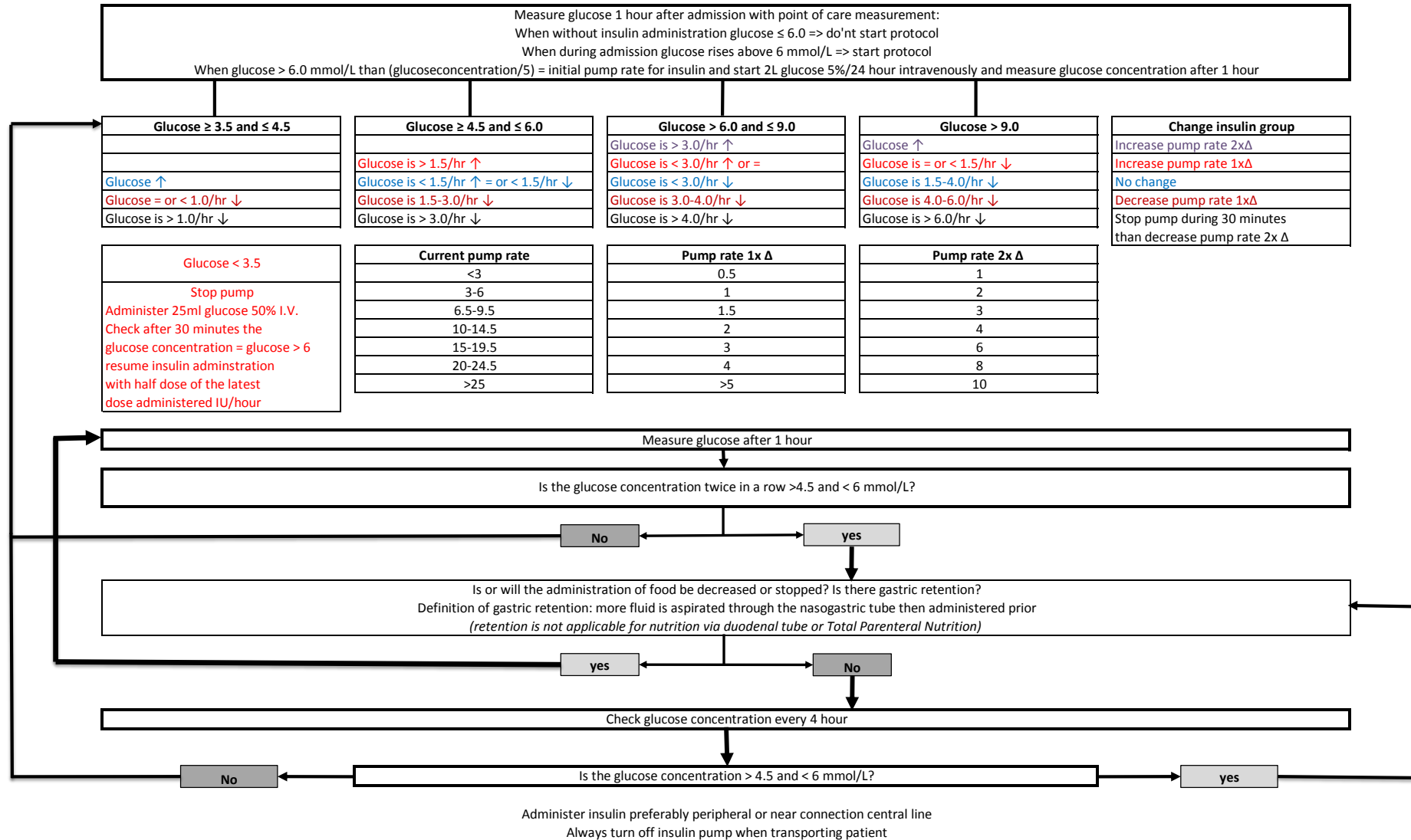

Supplement: Additional file 1: — Details of the tight glucose protocol. [file 13054_2015_959_MOESM1_ESM.pdf]
